# Notes and News

**Published:** 1901-02-09

**Authors:** 


### NOTES AND NEWS.

HOSPITAL MEETINGS.

City of London Lying-in Hospital.—Annual general meeting, Wednesday, February 13th, at 4.30 p.m., at the Hospital.

Hospital for Throat, Golden Square.—Annual general meeting, Wednesday, February 13th, at 3.30 p.m., at the Hospital.

St. Mark's Hospital.—Annual general meeting, Thursday, February 14th, at 12 noon, at the Mansion House.

A British Congress on Tuberculosis is to be held this year, and the King will open it at the Queen's Hall. Not only is Great Britain with her Colonies and Dependencies to be represented, but scientific representatives are to be invited from all parts of the world. The object of the congress will be to exchange information and experience on the subject of the disease. A museum will be one of the features of the congress. One special section of the congress will be devoted to the subject of tuberculosis in animals. Communications should be addressed to the Secretary-General, 20 Hanover Square.

The German International Congress will be held at Berlin in April.

The Prince of Wales's Hospital Fund has again been the recent fortunate recipient of a legacy. The late Mrs. Pate has bequeathed to it the sum of £1,000.

The outbreak of small-pox at Glasgow has been kept well in hand, and the sanitary officers have found ready co-operation from the inhabitants of the city. Nevertheless the number of patients is large, amounting to 433 in hospital.

Professor Schafer, Jodrell Professor of Physiology at University College, has received recognition for his services from his pupils and colleagues in the shape of a silver bowl and platters, and the endowment of a medal to be called after him, and to be an award for research in Physiology.

The Arbroath and District Hospital which is to be erected at Hercules Den just outside the city will accommodate 35 patients. Provision will be made for 18 scarlet fever, 10 typhoid, and five diphtheria cases, all in separate pavilions. An observation ward and the usual offices will complete the institution which is to cost about £13,000.

The late Mr. George Branston, of Newark-on-Trent, has bequeathed £1,000 to the Newark Town and County Hospital.

A Special Court of the Governors of the Middlesex Hospital has been held, at which an address was framed to present to the King.

The recent report of Sir W. Crookes and Professor Dewar upon the improved purity of London drinking-water is certainly a matter for general satisfaction.

Observations on the circumstances attendant on the visitations of bubonic plague in Hong Kong, show that the epidemic has abated when the temperature showed a record above 80 degrees Fahrenheit.

The Fleming Memorial Hospital for Sick Children at Newcastle is one of the very first institutions to found a memorial to Queen Victoria. At the recent annual meeting of the hospital it was decided to endow a cot as a special memorial, and contributions were at once received towards the scheme.

Two important hospitals — the Seaman's Hospital, Greenwich, and Addenbrooke's Hospital, Cambridge, have recently added their testimony to the beneficial results from the adoption of the out-door treatment for consumption, both having employed the method in the treatment of their patients.

New dangers from electricity have been revealed by the errible mishap at Liverpool. Some telephone wires gave way with the weight of the snow and fell across the overhead electric wires of the tramways. As pedestrians came in contact with the wires in the darkness they experienced shocks of more or less violence, according to whether the contact was direct with the wires or the current conducted by the snow. From some sufferers flames literally darted, and these were in terrible agony. The patients were conveyed to the Royal Infirmary.

At a recent Court of Governors held at St. George's Hospital it was decided to appoint a Deputy Treasurer and two Joint Treasurers, to be elected for life. The Deputy Treasurer is to be elected from amongst the Governors by a Board, and to hold office for five years, subject to re-election. It was also arranged to establish a Committee to investigate complaints, to consist of eleven members, consisting of the Treasurers and Deputy Treasurer, the Senior Physician and Senior Surgeon, the Dean of the Medical School, and five Governors. Various other important new regulations were made and existing institutions altered.

The Sanatinary Maritime and Quarantine Council of Egypt are seeking a medical officer to superintend the sanitation of Suez. Applications will be received up to March 9th, addressed to the Présidence du Conseil Quarantenaire at Alexandria. The applications must be accompanied by a copy of a diploma of Doctor of Medicine of a recognised university; testimony as to practice in bacteriology; of special proficiency in epidemiology; a certificate of health; and a formal undertaking, if nominated, that the candidate will present himself in Egypt during the month following the official announcement. The salary is 8,000 fr. per annum, advancing to 12,000 fr.

